# Test for Hyperchlorhydria

**Published:** 1913-06

**Authors:** 


TEST FOR HYPERCHLORHYDRIA. *La Tribune Medicale.* Cippollina claims when an aqueous solution of anilin is added to a solution containing HCl and a few drops of sodium hyperchlorite are added, the mixture assumes a color which varies with the intensity of the HCl. If it contains more than 2 per cent. of HCl a permanent amethyst color results. If the amount present exceeds ¼ per cent., a reddish-violet color changing to yellow appears; if less than ¼ per cent., a permanent dark violet color appears. By this means the hydrochloric acid of the gastric contents can be determined in a few minutes.

The test meal used by the author consists of 200 grm. of potato and 200 c.c. of white wine and water in the proportion of 1:2. A yellow reaction proves that hyperchlorhydria can be excluded. The same color is obtained with lactic acid, but only in 10 per cent. solutions.

A 10 per cent. solution of calcium hyperchlorite may be substituted for the sodium solution.

NOTE.—Probably hypochlorite is meant.

